# Supplementary material for: Epithelial disruption drives mesendoderm differentiation in human pluripotent stem cells by enabling TGF-β protein sensing
Source: Nat Commun. 2023 Jan 21;14:349. doi: 10.1038/s41467-023-35965-8 (PMC9867713; doi:10.1038/s41467-023-35965-8)
Supplement: Supplementary file 1 — Supplementary Information [file 41467_2023_35965_MOESM1_ESM.pdf]

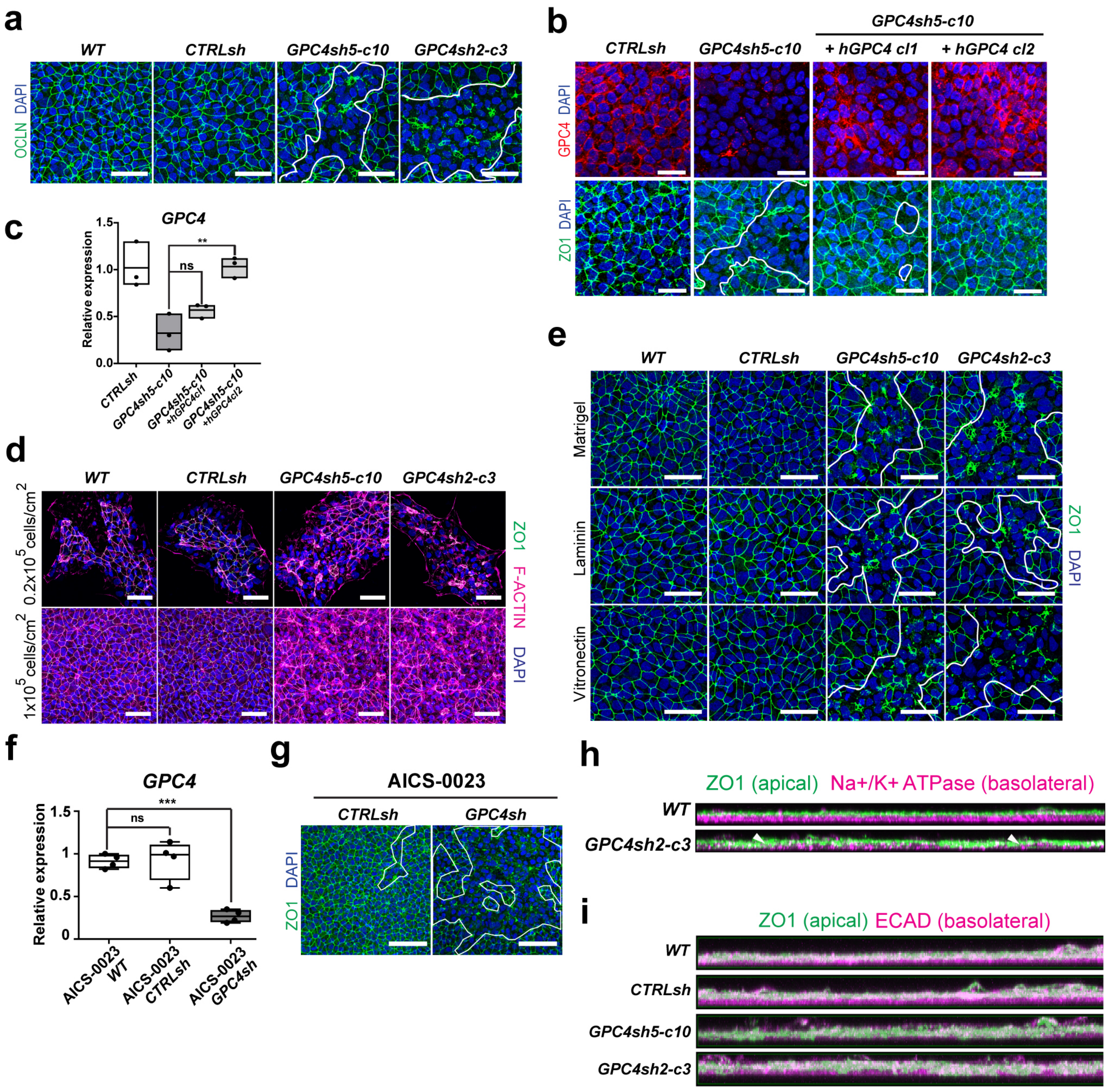
a
WT
CTRLsh
GPC4sh5-c10
GPC4sh2-c3
OCLN DAPI
b
GPC4sh5-c10
CTRLsh
GPC4sh5-c10
+ hGPC4 cl1
+ hGPC4 cl2
GPC4 DAPI
ZO1 DAPI
c
GPC4
Relative expression
1.5
1.0
0.5
0.0
ns
**
CTRLsh
GPC4sh5-c10
GPC4sh5-c10 +hGPC4cl1
GPC4sh5-c10 +hGPC4cl2
d
WT
CTRLsh
GPC4sh5-c10
GPC4sh2-c3
0.2x10^5 cells/cm^2
1x10^5 cells/cm^2
ZO1
F-ACTIN
DAPI
e
WT
CTRLsh
GPC4sh5-c10
GPC4sh2-c3
Matrigel
Laminin
Vitronectin
ZO1
DAPI
f
GPC4
Relative expression
1.5
1
0.5
0
ns
***
AICS-0023 WT
AICS-0023 CTRLsh
AICS-0023 GPC4sh
g
AICS-0023
CTRLsh
GPC4sh
ZO1 DAPI
h
ZO1 (apical) Na+/K+ ATPase (basolateral)
WT
GPC4sh2-c3
i
ZO1 (apical) ECAD (basolateral)
WT
CTRLsh
GPC4sh5-c10
GPC4sh2-c3

Supplementary Fig. 1. Down-regulation of GPC4 affects hiPSC epithelial integrity by disrupting TJs. (a) Immunofluorescence analysis of OCLN (green) in WT, CTRLsh, GPC4sh5-c10 and GPC4sh2-c3 029 hiPSCs showing extensive zones of disrupted TJs in GPC4sh hiPSCs, n=1. Scale bar: 50 μm. **(b)** Immunofluorescence analysis of GPC4 (red), ZO1 (green) and DAPI positive nuclei (blue) in CTRLsh, GPC4sh5-c10, GPC4sh5-c10+hGPC4cl1 and GPC4sh5-c10+hGPC4cl2 O29 hiPSCs, n=3. Scale bar: 50 μm. **(c)** RT-qPCR analyses of *GPC4* transcript levels in CTRLsh, GPC4sh5-c10, GPC4sh5-c10+hGPC4cl1 and GPC4sh5-c10+hGPC4cl2 029 hiPSC lines showing the *GPC4* expression rescue in GPC4sh5-c10 hiPSCs. Box plots represent the mean with min and max values, n=3. Note the correlation between GPC4 levels in cells and the rescue of epithelial disruption. **(d)** Immunofluorescence of F-ACTIN (magenta), ZO1 (green) and DAPI (blue) in WT, CTRLsh, GPC4sh5-c10 and GPC4sh2-c3 029 hiPSCs cultured at density of $0.2x10^5$ cells/cm^2, and $1x10^5$ cells/cm^2, n=1 Scale bar: 50 μm. **(e)** Immunofluorescence analysis of ZO1 (green) and DAPI (blue) in WT, CTRLsh, GPC4sh5-c10 and GPC4sh2-c3 029 hiPSCs cultured on Matrigel, Laminin and Vitronectin, n=3. Scale bar: 50 μm. **(f)** RT-qPCR analyses of *GPC4* transcript levels in WT, CTRLsh and GPC4sh AICS-0023 hiPSC lines showing a down-regulation of *GPC4* expression in GPC4sh hiPSCs. Box plots represent the mean with interquartile range, the errors bars indicate min and max values, n=4. **(g)** Immunofluorescence analysis of ZO1 (green) and DAPI (blue) in WT, CTRLsh and GPC4sh AICS-0023 hiPSC showing extensive zones of disrupted TJs in GPC4sh hiPSCs, n=2. Scale bar: 50 μm. **(h)** Immunofluorescence analysis of ZO1 (green) and Na^+/K^+ ATPase (magenta) in WT and GPC4sh2-c3 029 hiPSCs. Pictures are presented as lateral cell view, n=3. **(i)** Immunofluorescence analysis of ZO1 (green) and E-CAD (magenta) in WT, CTRLsh,

GPC4sh5-c10 and GPC4sh2-c3 029 hiPSCs. Pictures are presented as lateral cell view, n=3. Statistical analysis for the overall figure: (c, f) one-way ANOVA followed by Dunnett's multiple comparison test. P values: (***) < 0.001, (**) < 0.01, (*) < 0.05, ns= not significant. For all panels "n" corresponds to the number of biological replicates. Source data are provided as a Source Data file.

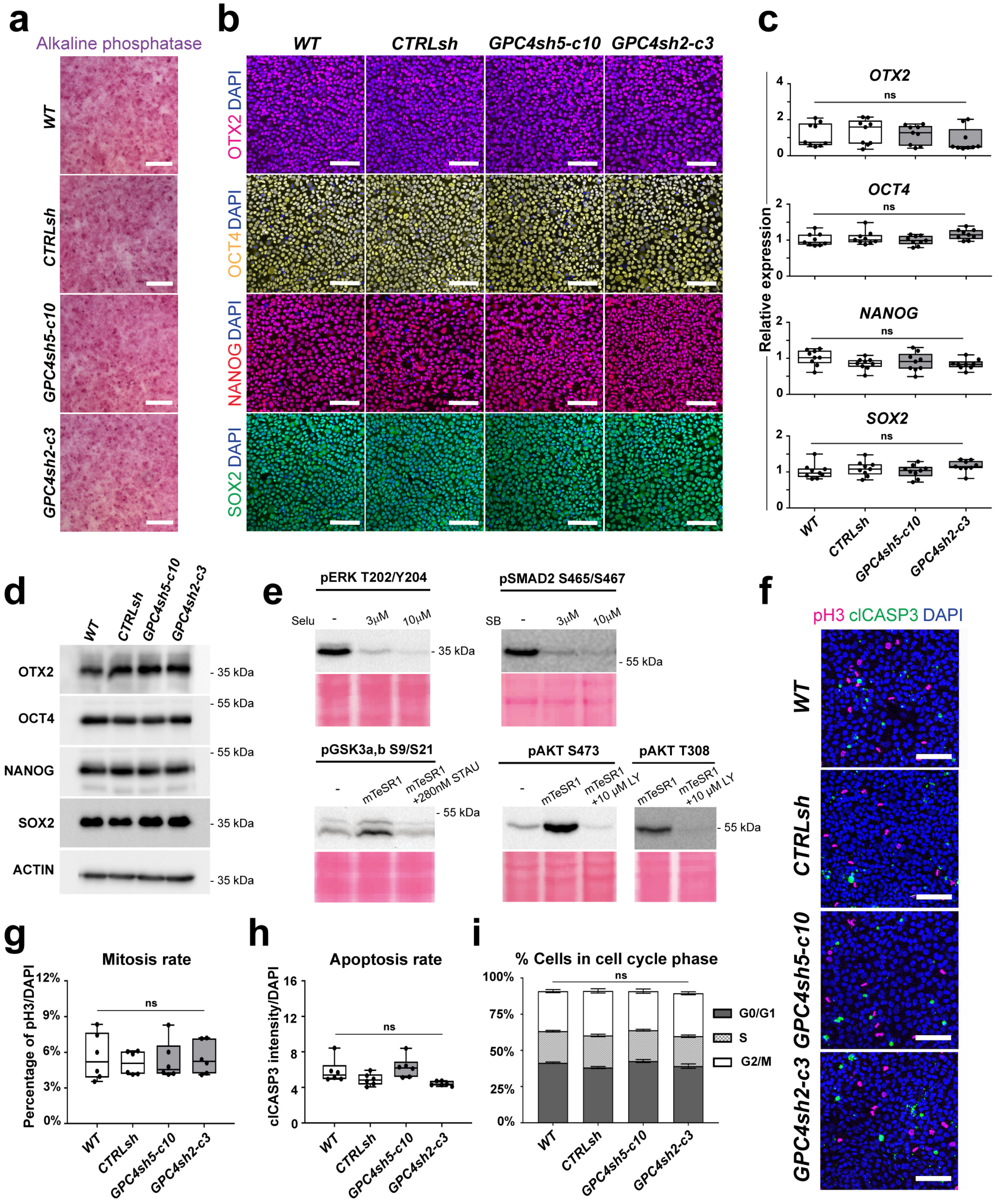
a
Alkaline phosphatase
WT
CTRLsh
GPC4sh5-c10
GPC4sh2-c3
b
WT
CTRLsh
GPC4sh5-c10
GPC4sh2-c3
OTX2 DAPI
OCT4 DAPI
NANOG DAPI
SOX2 DAPI
c
Relative expression
OTX2
OCT4
NANOG
SOX2
ns
d
OTX2
OCT4
NANOG
SOX2
ACTIN
- 35 kDa
- 55 kDa
e
pERK T202/Y204
pSMAD2 S465/S467
Selu
SB
3μM
10μM
pGSK3a,b S9/S21
pAKT S473
pAKT T308
mTeSR1
mTeSR1 +280nM STAU
mTeSR1 +10 μM LY
f
pH3 clCASP3 DAPI
g
Mitosis rate
Percentage of pH3/DAPI
h
Apoptosis rate
clCASP3 intensity/DAPI
i
% Cells in cell cycle phase
G0/G1
S
G2/M

Supplementary Fig. 2. Epithelial integrity disruption does not affect stemness. (a) Alkaline phosphatase staining of the indicated 029 hiPSCs, n=1. Scale bar:100 μm. **(b)** Immunofluorescence of OTX2 (magenta) OCT4 (yellow), NANOG (red) and SOX2 (green) in the indicated 029 hiPSCs, n=3. Scale bar:100 μm. **(c)** RT-qPCR analysis of *OTX2*, *OCT4*, *NANOG* and *SOX2* in the indicated 029 hiPSCs. Box plots represent the median with interquartile range, errors bars indicate min and max values, n=9. **(d)** Protein extract of the indicated 029 hiPSCs analyzed by western-blot with OTX2, OCT4, NANOG, and SOX2 antibodies, n=3. Blots were processed in parallel. **(e)** Western-blots of pERK T202/Y204, pSMAD2 S465/S467, pGSK3a, b S9/S21 pAKT S473, and pAKT T308 in CTRLsh hiPSCs to test antibody specificity. For pERK T202/Y204 and pSMAD2 S465/S467, CTRLsh hiPSCs were untreated or treated with 3μM/10μM of Selumetinib (Selu; Tocris) or SB-431542 (SB; Tocris) at 3μM/10μM, respectively. For pGSK3a,b S9/S21, and pAKT S473, pAKT T308, starved CTRLsh hiPSCs were stimulated with mTeSR1 for 15 min in the presence or not of 280nM Stauroporine (STAU; Sigma) or of 10 μM LY294002 (LY; Selleck), respectively. Membrane ponceau staining was used as loading control, n=2. **(f)** Immunofluorescence analysis of pH3 (magenta) and cl-CASP3 (green) in indicated 029 hiPSCs. Scale bar: 100 μm, n=3. **(g)** Percentages of pH3 positive cells quantified from staining in (f). Box plots represent the median with interquartile range, whiskers indicate min and max values, n=3. **(h)** Cl-CASP3 global intensities quantified from staining in (f). **(i)** Quantification of the cell percentage in the cell cycle phases G0/G1, S and G2/M. Data are represented as stacked bars with the mean ± SEM, n=6. Box plots represent the median with interquartile range, whiskers indicate min and max values, n=3. Statistical analysis for the overall figure: (c, g, h) one-way ANOVA followed by Dunnett's multiple comparison test or (i) two-way ANOVA followed by

Tukey's multiple comparison tests. P values: (***) < 0.001, (**) < 0.01, (*) < 0.05, ns= not significant. For all panels "n" corresponds to the number of biological replicates. Source data are provided as a Source Data file.

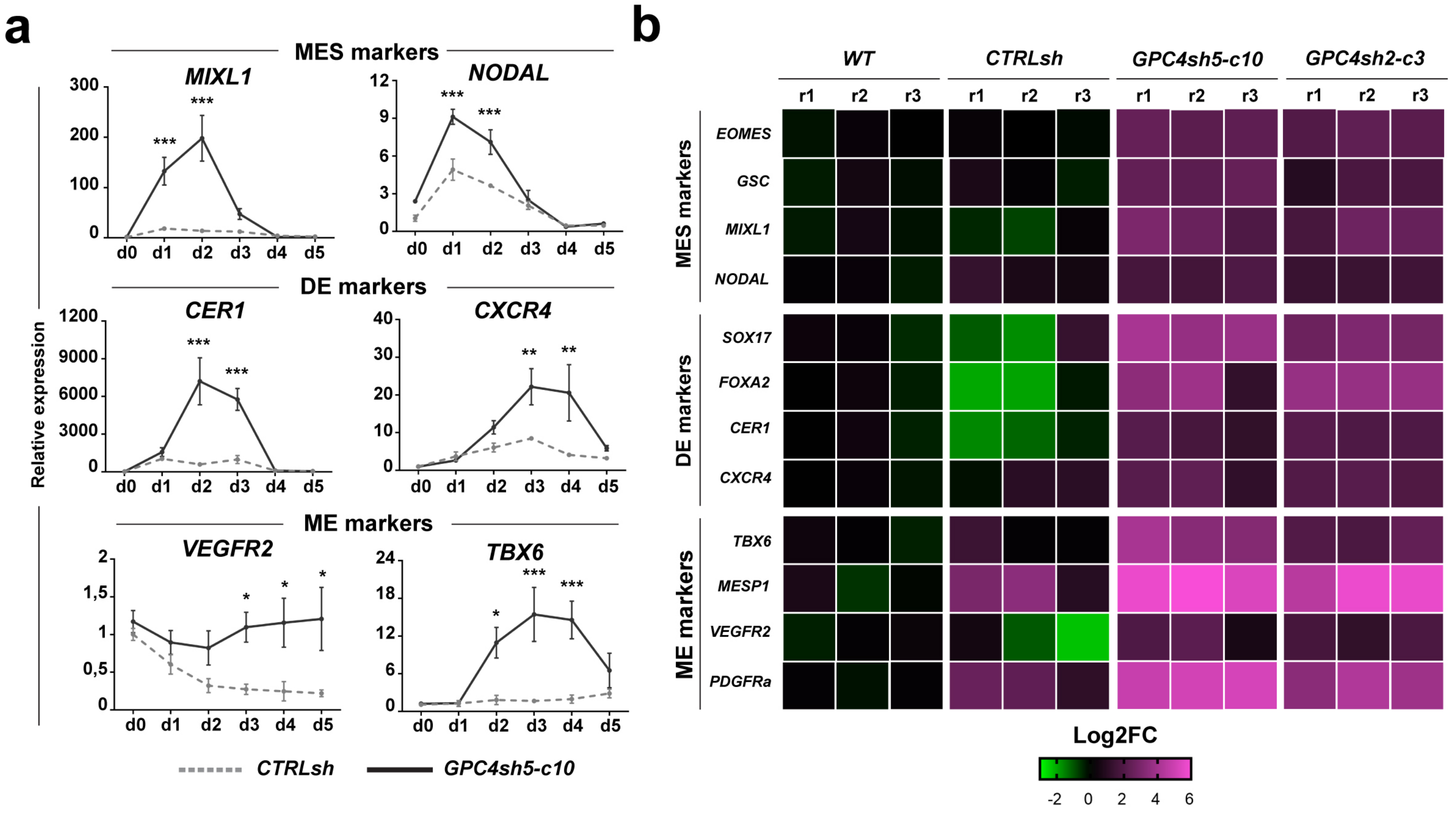
a
MES markers
MIXL1
NODAL
DE markers
CER1
CXCR4
ME markers
VEGFR2
TBX6
Relative expression
d0 d1 d2 d3 d4 d5
CTRLsh
GPC4sh5-c10
b
WT
CTRLsh
GPC4sh5-c10
GPC4sh2-c3
r1 r2 r3
MES markers
EOMES
GSC
MIXL1
NODAL
DE markers
SOX17
FOXA2
CER1
CXCR4
ME markers
TBX6
MESP1
VEGFR2
PDGFRa
Log2FC
-2 0 2 4 6

Supplementary Fig. 3. Disruption of epithelial integrity enhances differentiation potential of hiPSCs. (a) CTRLsh and GPC4sh5-c10 029 hiPSCs were differentiated for 5 days into DE or ME and transcript levels of MES, DE or ME specific markers were subsequently analyzed by RT-qPCR. Top panel: MES markers (*MIXL1* and *NODAL*), middle panel: DE markers (*CER1* and *CXCR4*) and bottom panel: ME markers (*VEGFR2* and *TBX6*). Data were normalized to the d0 of CTRLsh and represented as mean ± SEM, n=3. **(b)** WT, CTRLsh, GPC4sh5-c10 and GPC4sh2-c3 029 hiPSCs were differentiated for 1 day into MES, 3 days into DE or 3 days into ME, and transcript levels of lineage specific markers were analyzed by RT-qPCR. Top panel: MES markers (*EOMES*, *GSC*, *MIXL1* and *NODAL*); middle panel: DE markers (*SOX17*, *FOXA2*, *CER1* and *CXCR4*), bottom panel: ME markers (*TBX6*, *MESP1*, *VEGFR2* and *PDGFRa*). Transcript levels were normalized to the mean of WT ΔCt, data are represented as heatmap of Log_2FC, n=3. Statistical analysis for the overall figure: two-way ANOVA followed by Sidak's multiple comparison test. P values: (***) < 0.001, (**) < 0.01, (*) < 0.05, ns= not significant. For all panels "n" corresponds to the number of biological replicates. Source data are provided as a Source Data file.

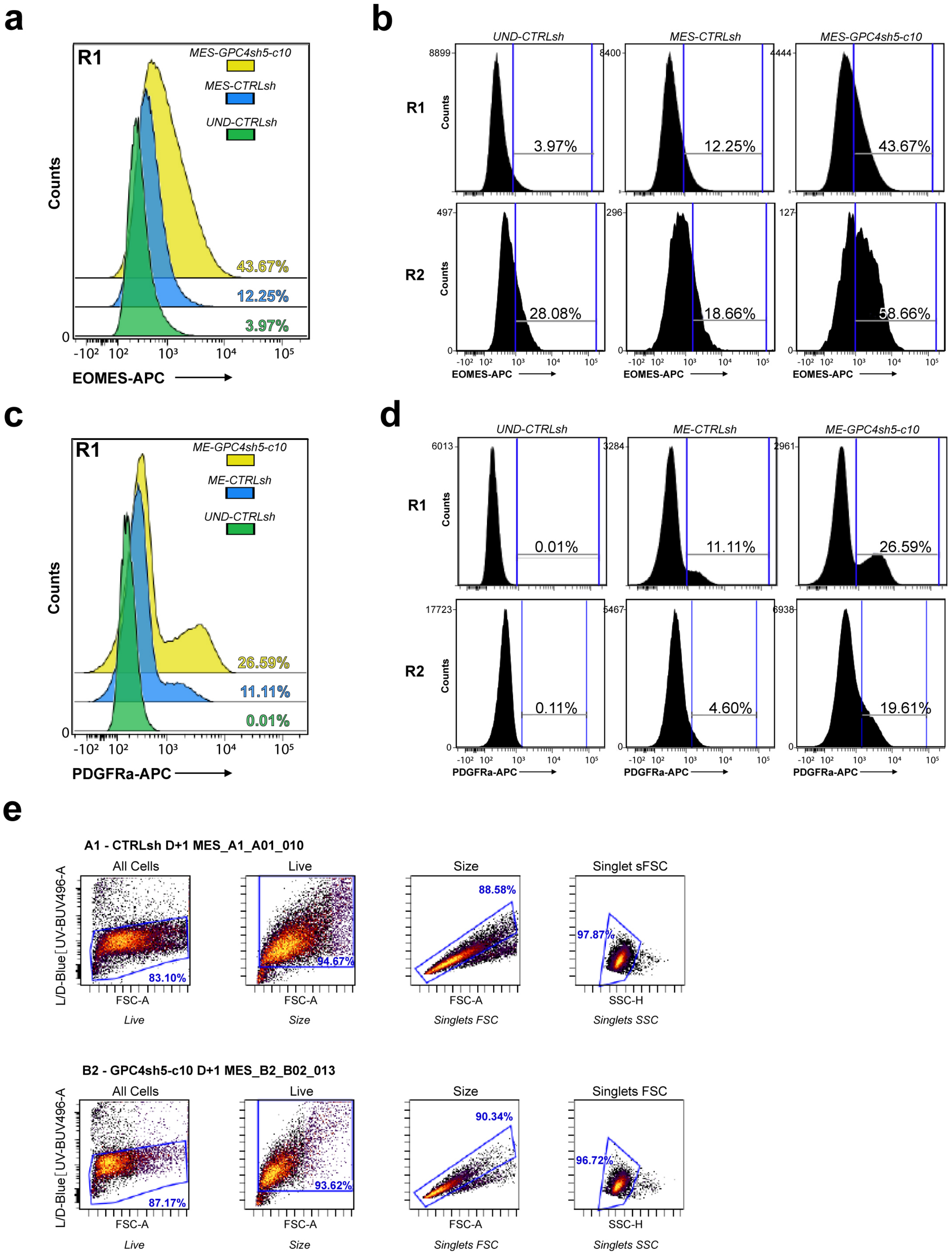
a
R1
MES-GPC4sh5-c10
MES-CTRLsh
UND-CTRLsh
Counts
43.67%
12.25%
3.97%
EOMES-APC
b
UND-CTRLsh
MES-CTRLsh
MES-GPC4sh5-c10
R1
R2
Counts
3.97%
12.25%
43.67%
28.08%
18.66%
58.66%
EOMES-APC
c
R1
ME-GPC4sh5-c10
ME-CTRLsh
UND-CTRLsh
Counts
26.59%
11.11%
0.01%
PDGFRa-APC
d
UND-CTRLsh
ME-CTRLsh
ME-GPC4sh5-c10
R1
R2
Counts
0.01%
11.11%
26.59%
0.11%
4.60%
19.61%
PDGFRa-APC
e
A1 - CTRLsh D+1 MES_A1_A01_010
All Cells
Live
Size
Singlet sFSC
L/D-Blue [UV-BUV496-A
83.10%
94.67%
88.58%
97.87%
FSC-A
SSC-H
Live
Size
Singlets FSC
Singlets SSC
B2 - GPC4sh5-c10 D+1 MES_B2_B02_013
All Cells
Live
Size
Singlets FSC
87.17%
93.62%
90.34%
96.72%

Supplementary Fig. 4. Disruption of epithelial integrity enhances differentiation potential of hiPSCs. (a) Histogram plot reporting the flow cytometry analysis of EOMES positive cells in hiPSCs differentiated along the mesendoderm lineage (MES). Flow cytometry profiles of differentiated CTRLsh and GPC4sh5 cells (MES-CTRLsh and MES-GPC4sh5, respectively), and of undifferentiated CTRLsh hiPSCs. Numbers correspond to the percentage of EOMES positive cells in each cell type. The histogram shows results from one biological replicate (indicated as R1 top left corner of the histogram plot). **(b)** Flow cytometry profiles of the individual samples in **(a)** and of those corresponding to a second biological replicate (R2). Numbers correspond to the percentage of EOMES positive cells in each cell type. **(c)** Histogram plot reporting the flow cytometry analysis of PDGFRa positive cells in hiPSCs differentiated along the mesoderm lineage (ME). Flow cytometry profiles of differentiated CTRLsh and GPC4sh5 cells (ME-CTRLsh and MES-GPC4sh5, respectively), and of undifferentiated CTRLsh hiPSCs. Numbers correspond to the percentage of PDGFRa positive cells in each cell type. Results are from one biological replicate (indicated as R1 top left corner of the histogram plot). **(d)** Flow cytometry profiles of the individual samples in **(c)** and of those corresponding to a second biological replicate (R2). Numbers correspond to the percentage of PDGFRa positive cells in each cell type. **(e)** Images reporting the gating strategies used for the above flow cytometry images. Gating for images (a) and (b) are shown as an example. All events shown in (a), (b), (c) and (d) were gated on Singlet FSC events. Source data are provided as a Source Data file.

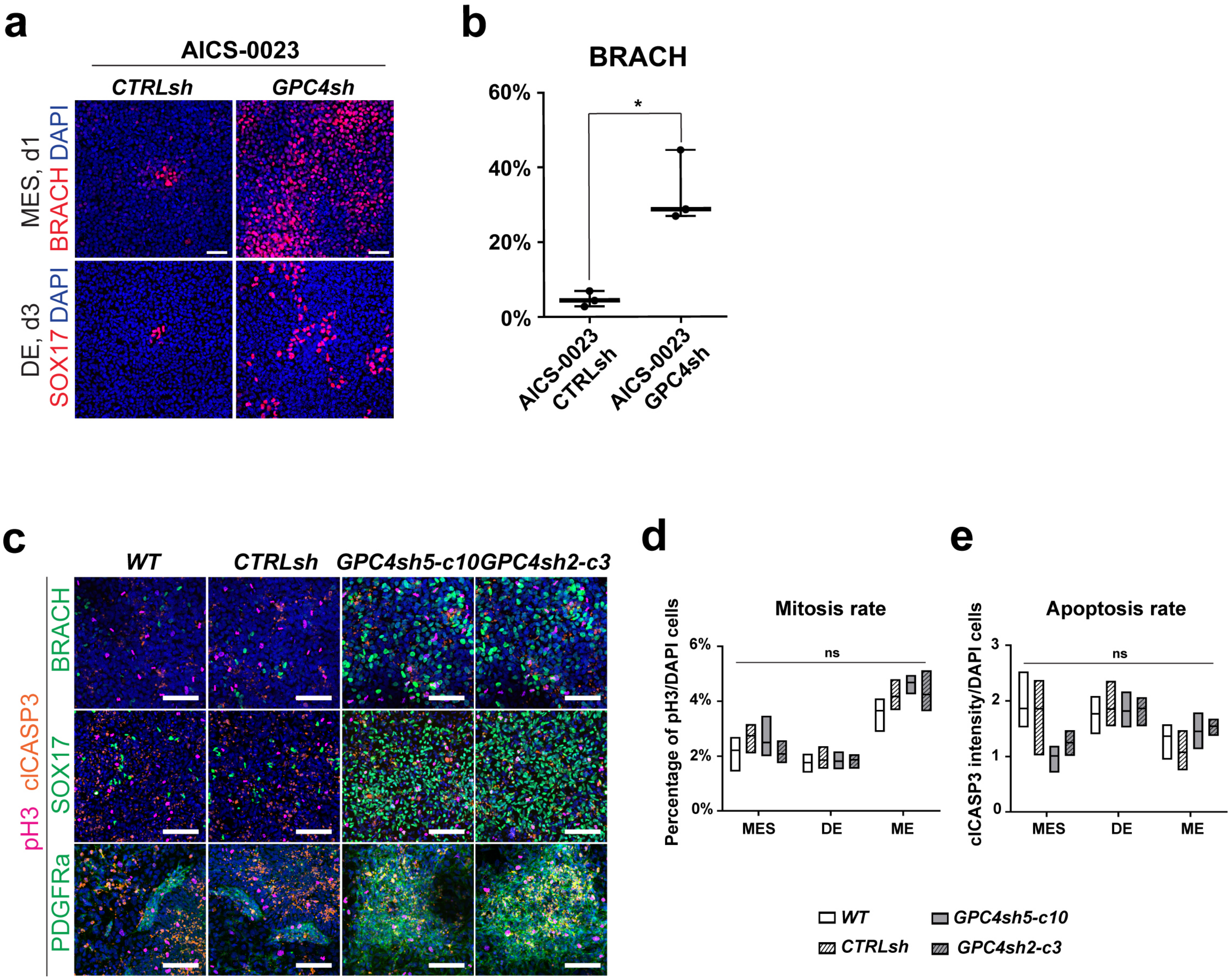
a
AICS-0023
CTRLsh
GPC4sh
MES, d1 BRACH DAPI
DE, d3 SOX17 DAPI
b
BRACH
60%
40%
20%
0%
*
AICS-0023 CTRLsh
AICS-0023 GPC4sh
c
WT
CTRLsh
GPC4sh5-c10
GPC4sh2-c3
pH3 clCASP3
BRACH
SOX17
PDGFRa
d
Mitosis rate
Percentage of pH3/DAPI cells
6%
4%
2%
0%
ns
MES
DE
ME
e
Apoptosis rate
clCASP3 intensity/DAPI cells
3
2
1
0
ns
MES
DE
ME
WT
CTRLsh
GPC4sh5-c10
GPC4sh2-c3

Supplementary Fig. 5. Analysis of cell differentiation properties in GPC4sh hiPSCs. (a) Immunofluorescence of the MES marker, BRACH (red), and of the DE marker, SOX17 (red), in CTRLsh and GPC4s AICS-0023 hiPSCs differentiated for 1 day into MES and 3 days into DE, n=3. Scale bar: 100 μm. **(b)** Percentages of BRACH positive cells were quantified from staining shown in (c). Box plots represent the median, the whiskers indicate min and max values, n=3. **(c)** Immunofluorescence analysis of pH3 (magenta), cl-CASP3 (orange) with BRACH (green, PS), SOX17 (green, DE) or PDGFRa (green, ME) in WT, CTRLsh, GPC4sh5-c10 and GPC4sh2-c3 029 hiPSCs differentiated for 1 day into PS, 3 days into DE or 3 days into ME. Scale bar: 100 μm, n=3. **(d)** Percentages of pH3 positive cells were quantified from staining shown in (c). Box plots represent the mean with min and max values, n=3. **(e)** Global intensities of cl-CASP3 were quantified from staining shown in (d). Box plots represent the mean with min and max values, n=3. Statistical analysis for the overall figure: (b, d, e) two-way ANOVA followed by Tukey's (d, e) multiple comparison tests. P values: (***) < 0.001, (**) < 0.01, (*) < 0.05, ns= not significant. For all panels "n" corresponds to the number of biological replicates. Source data are provided as a Source Data file.

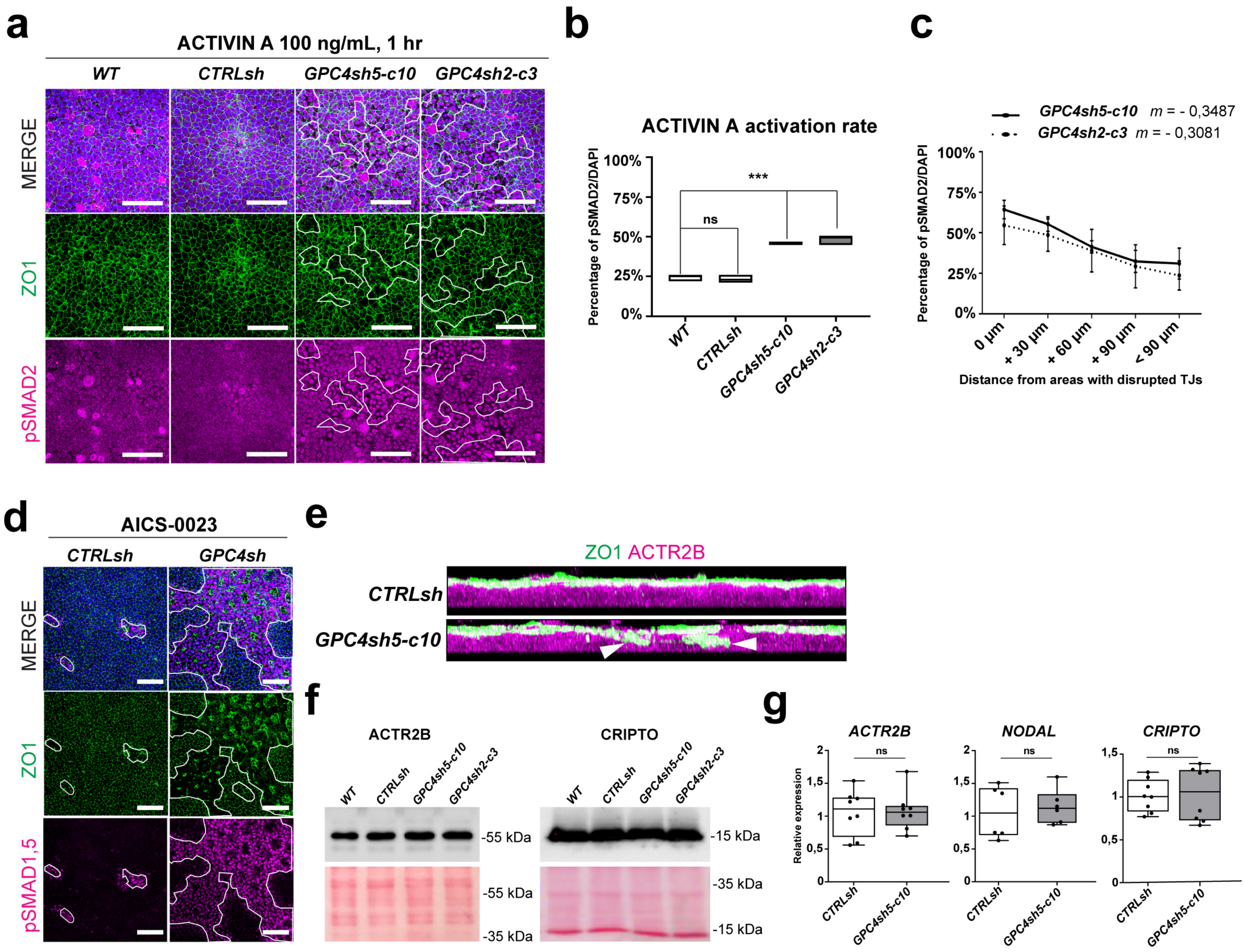
a
ACTIVIN A 100 ng/mL, 1 hr
WT
CTRLsh
GPC4sh5-c10
GPC4sh2-c3
MERGE
ZO1
pSMAD2
b
ACTIVIN A activation rate
Percentage of pSMAD2/DAPI
100%
75%
50%
25%
0%
***
ns
WT
CTRLsh
GPC4sh5-c10
GPC4sh2-c3
c
GPC4sh5-c10 m = - 0,3487
GPC4sh2-c3 m = - 0,3081
Percentage of pSMAD2/DAPI
100%
75%
50%
25%
0%
0 µm
+ 30 µm
+ 60 µm
+ 90 µm
< 90 µm
Distance from areas with disrupted TJs
d
AICS-0023
CTRLsh
GPC4sh
MERGE
ZO1
pSMAD1,5
e
ZO1 ACTR2B
CTRLsh
GPC4sh5-c10
f
ACTR2B
CRIPTO
WT
CTRLsh
GPC4sh5-c10
GPC4sh2-c3
-55 kDa
-55 kDa
-35 kDa
-15 kDa
-35 kDa
-15 kDa
g
ACTR2B
NODAL
CRIPTO
Relative expression
ns
2
1,5
1
0,5
0
CTRLsh
GPC4sh5-c10

Supplementary Fig. 6. Epithelial integrity regulates TGF-β signaling response and patterning. (a) Immunofluorescence analysis of pSMAD2 (magenta) and ZO1 (green) of WT, CTRLsh, GPC4sh5-c10 and GPC4sh2-c3 029 hiPSCs stimulated for 1 hour with ACTIVIN A (100 ng/ml). Dashed areas: zones of disrupted TJs, n=3. Scale bar:50 μm. **(b)** Percentages of pSMAD2 positive cells quantified from staining in (a). Box plots represent the mean with min and max values, n=3. **(c)** Percentages of pSMAD2 positive cells as a function of distance from zones of disrupted TJs were quantified in GPC4sh5-c10 and GPC4sh2-c3 029 hiPSCs from staining in (a). 0 μm indicates cells located within zones of disrupted TJs, +30 μm, +60 μm, +90 μm and > +90 μm indicate cells located at 0 to 30μm, 30 to 60μm, 60 to 90μm or more than 90μm away from zones of disrupted TJs, respectively. Data are represented as mean ± SD, n=3. **(d)** Immunofluorescence analysis of pSMAD1,5 (magenta) and ZO1 (green) in CTRLsh and GPC4sh AICS-0023 hiPSCs stimulated for 1 hour with BMP4 (50 μg/mL). Dashed areas: zones of disrupted TJs, n=1. Scale bar:100 μm. **(e)** Immunofluorescence analysis of ZO1 (green) and ACTR2B (magenta), in CTRLsh and GPC4sh5-c10 029 hiPSCs. Pictures are presented as lateral view. Arrow heads point to areas of abnormal apical-basal polarity, n=2. **(f)** Total protein extract of WT, CTRLsh, GPC4sh5-c10 and GPC4sh2-c3 029 hiPSCs were analyzed by Western-blot with ACTR2B and CRIPTO antibodies. Membrane ponceau staining was used as loading control, n=2. **(g)** RT-qPCR analysis of transcript levels of *ACTR2B*, *NODAL* and *CRIPTO* in CTRLsh and GPC4sh5-c10 029 hiPSCs. Data are represented as box and whisker plot, n=8 (*ACTR2B* and *CRIPTO*) or 6 (*NODAL*). Box plots represent the median with interquartile range, whiskers indicate min and max values, n=3. Statistical analysis for the overall figure: (b) one-way ANOVA followed by Dunnett's multiple comparison tests, (c) linear regression analysis and (g) paired t-

test analysis. P values: (***) < 0.001, (**) < 0.01, (*) < 0.05, ns= not significant. For all panels “n” corresponds to the number of biological replicates. Source data are provided as a Source Data file.

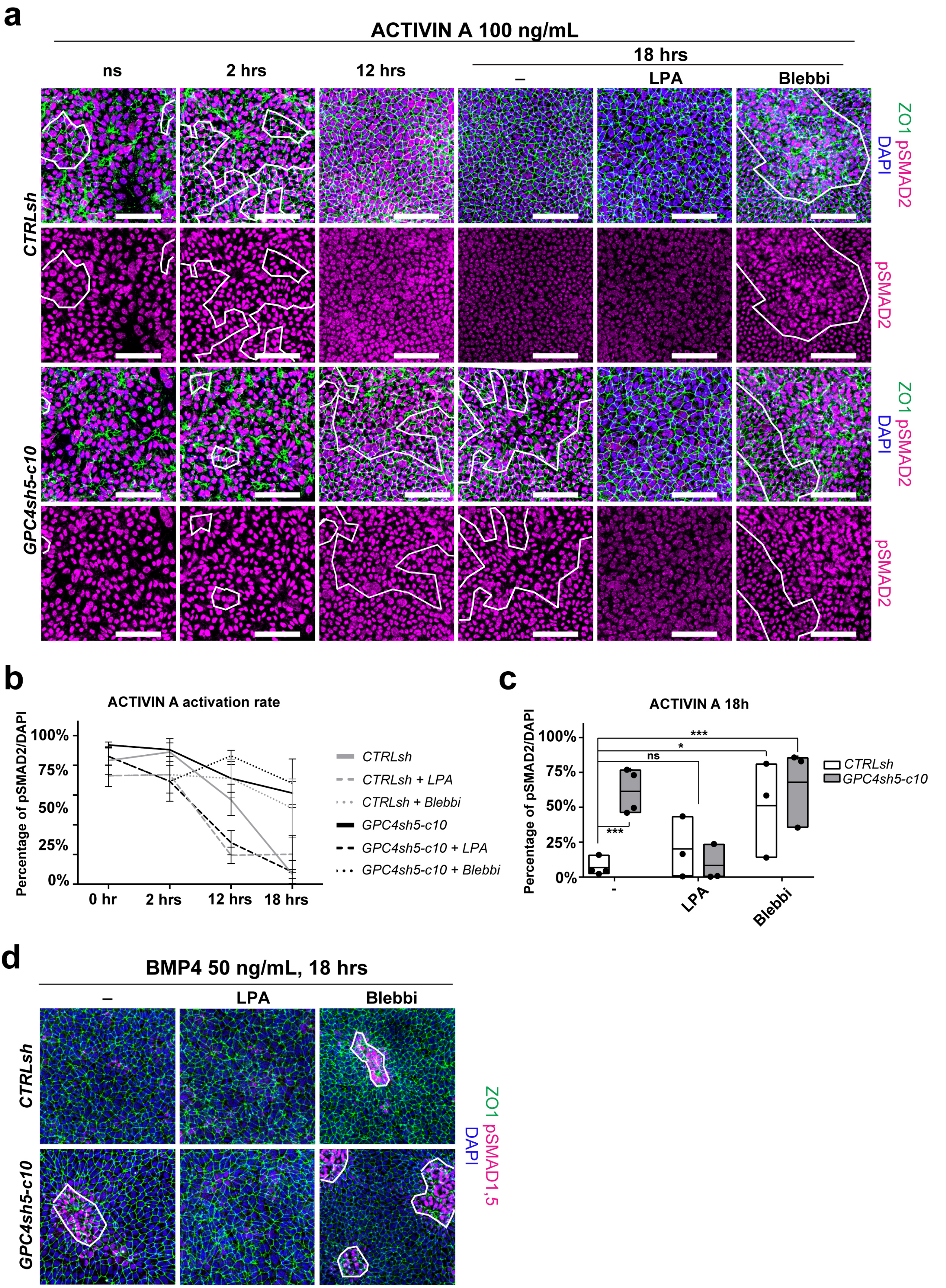
a
ACTIVIN A 100 ng/mL
18 hrs
ns
2 hrs
12 hrs
–
LPA
Blebbi
CTRLsh
GPC4sh5-c10
ZO1 pSMAD2 DAPI
pSMAD2
b
ACTIVIN A activation rate
Percentage of pSMAD2/DAPI
100%
75%
50%
25%
0%
0 hr
2 hrs
12 hrs
18 hrs
CTRLsh
CTRLsh + LPA
CTRLsh + Blebbi
GPC4sh5-c10
GPC4sh5-c10 + LPA
GPC4sh5-c10 + Blebbi
c
ACTIVIN A 18h
***
*
ns
***
-
LPA
Blebbi
CTRLsh
GPC4sh5-c10
d
BMP4 50 ng/mL, 18 hrs
–
LPA
Blebbi
CTRLsh
GPC4sh5-c10
ZO1 pSMAD1,5 DAPI

Supplementary Fig. 7. Disruption of epithelial integrity prolongs TGF-β signaling activation. (a) Immunofluorescence analysis of pSMAD2 (magenta) and ZO1 (green) proteins in low densities cultures of CTRLsh and GPC4sh5-c10 029 hiPSCs stimulated for 2, 12 and 18 hours with 100 ng/mL of ACTIVIN A with or without addition of LPA and Blebbi after 6 hours of stimulation, n=4 (normal condition) or 3 (LPA and Blebbi condition). Scale bar: 100 μm. **(b)** Percentages of pSMAD2 expressing cells were quantified from staining shown in (a). Data are represented as mean ± SEM, n=4 (normal condition) or 3 (LPA and Blebbi condition). **(c)** Percentages of pSMAD2 expressing cells for the time point 18 hours from (a-b). Box plots represent the mean with min and max values, n=3. **(d)** Immunofluorescence analysis of pSMAD1,5 (magenta) and ZO1 (green) proteins after 18 hours of BMP4 stimulation at 50 ng/mL with or without addition of LPA and Blebbi after the first 6 hours of stimulation, n=3. Scale bar: 100 μm. Statistical analysis for the overall figure: two-way ANOVA followed by Tukey's multiple comparison test. P values: (***) < 0.001, (**) < 0.01, (*) < 0.05, ns= not significant. For all panels "n" corresponds to the number of biological replicates. Source data are provided as a Source Data file.

Supplementary Table 1: Genes analysed for the Heatmap epithelial vs mesenchymal in Figure 1e

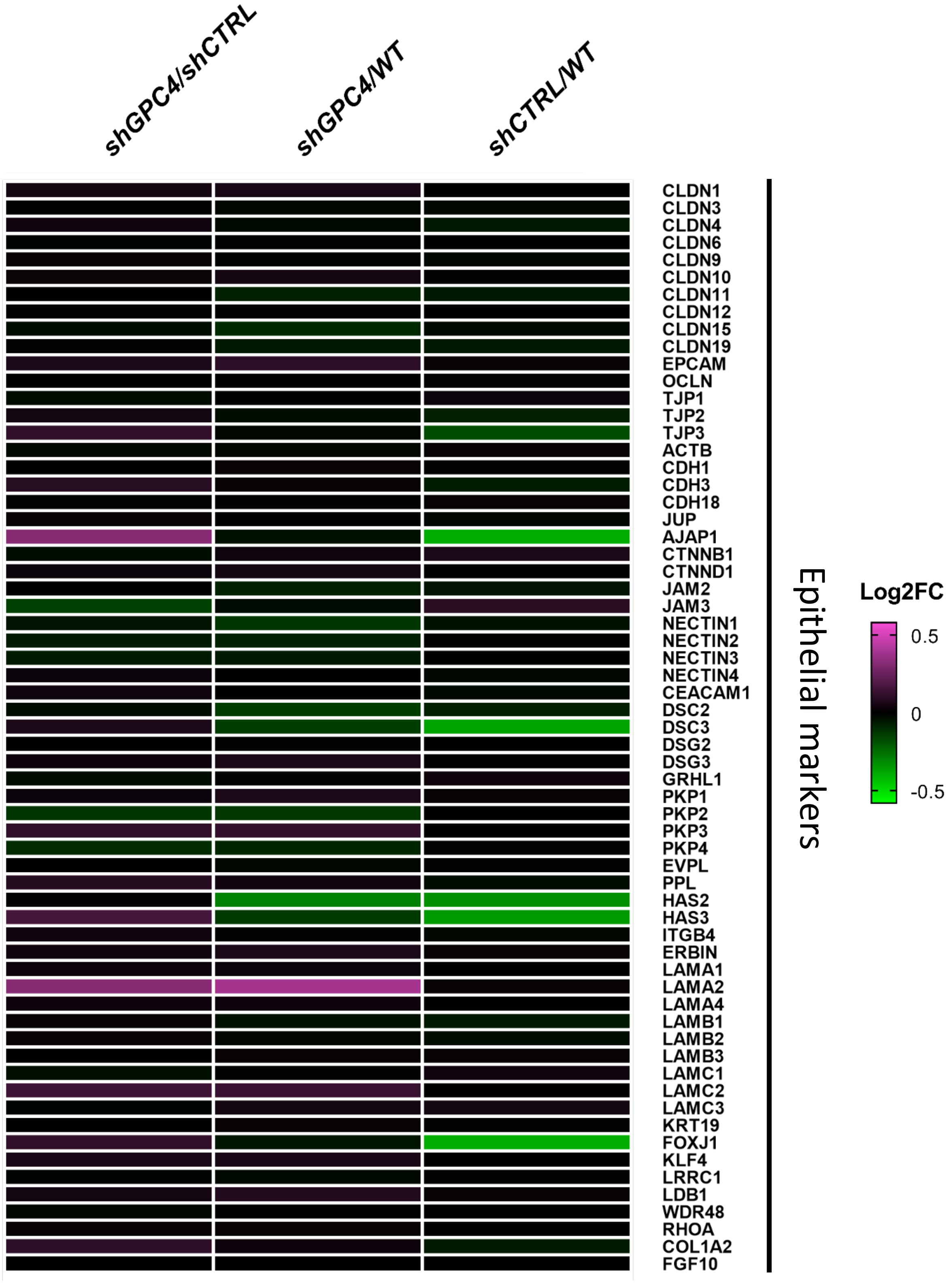

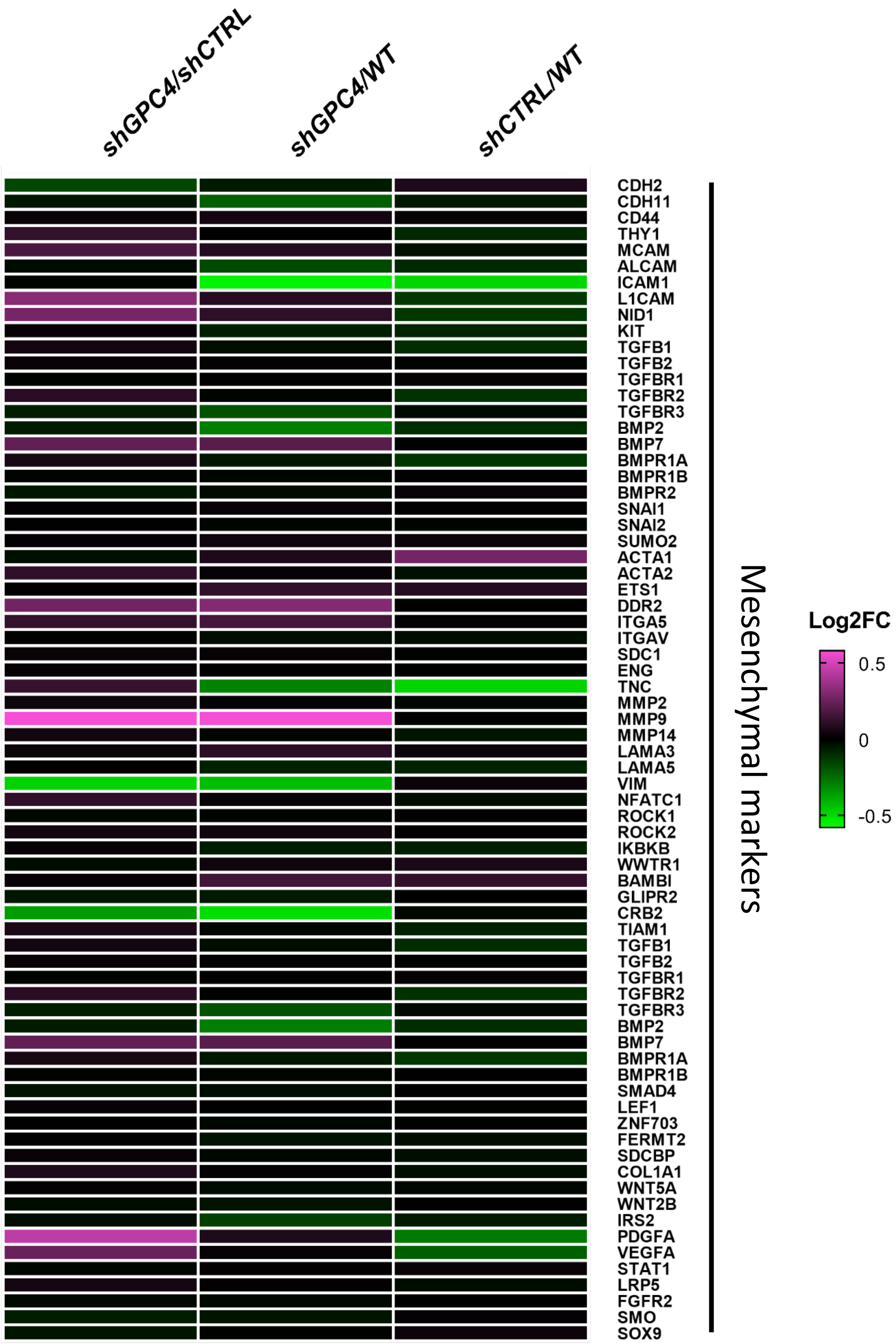
shGPC4/shCTRL
shGPC4/WT
shCTRL/WT
CDH2
CDH11
CD44
THY1
MCAM
ALCAM
ICAM1
L1CAM
NID1
KIT
TGFB1
TGFB2
TGFBR1
TGFBR2
TGFBR3
BMP2
BMP7
BMPR1A
BMPR1B
BMPR2
SNAI1
SNAI2
SUMO2
ACTA1
ACTA2
ETS1
DDR2
ITGA5
ITGAV
SDC1
ENG
TNC
MMP2
MMP9
MMP14
LAMA3
LAMA5
VIM
NFATC1
ROCK1
ROCK2
IKBKB
WWTR1
BAMBI
GLIPR2
CRB2
TIAM1
TGFB1
TGFB2
TGFBR1
TGFBR2
TGFBR3
BMP2
BMP7
BMPR1A
BMPR1B
SMAD4
LEF1
ZNF703
FERMT2
SDCBP
COL1A1
WNT5A
WNT2B
IRS2
PDGFA
VEGFA
STAT1
LRP5
FGFR2
SMO
SOX9
Mesenchymal markers
Log2FC
0.5
0
-0.5

Supplementary Table 2: Genes analysed for the Heatmap Pluripotency in Figure 2a

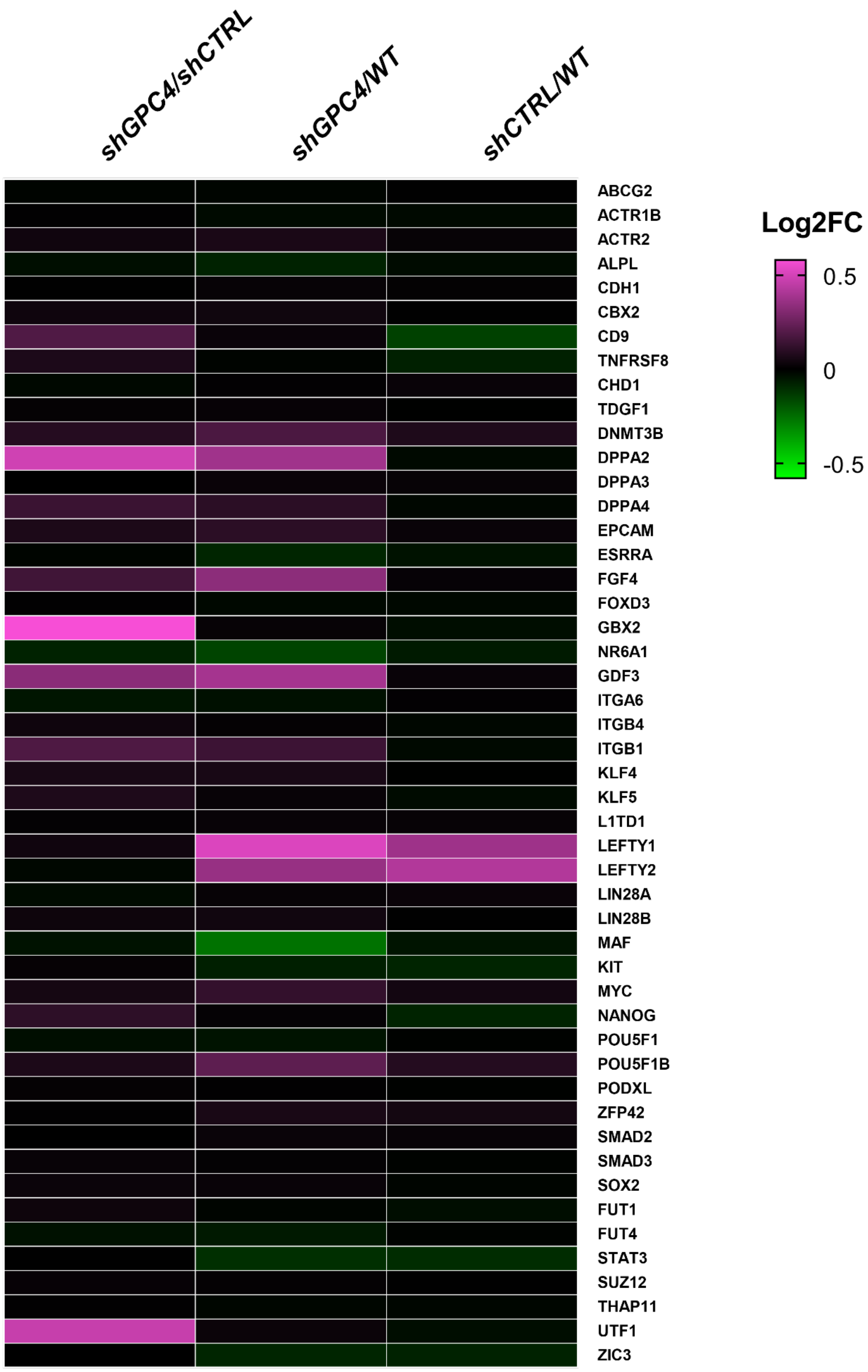

Supplementary Table 3. Protocols for hiPSC differentiation into MES, DE and ME

<table>
<tr><th></th><th>Day -1</th><th>Day 0</th><th>Day 1</th><th>Days 2 & 3</th><th>Days 4 & 5</th></tr>
<tr><th>MES</th><td rowspan="3">mTeSR1
+ Ri</td><td rowspan="3">mTeSR1</td><td rowspan="3">DE medium
+ ActivinA
(100ng/mL)</td><td colspan="2">-</td></tr>
<tr><th>DE</th><td>DE medium
+ 0,2% FBS
+ ActivinA
(100ng/mL)</td><td>DE medium
+ 2% FBS</td></tr>
<tr><th>ME</th><td>ME medium
+ Bmp4 (10ng/mL)</td><td>ME medium</td></tr>
</table>

Supplementary Table 4. Protocols for rescue and chemical induction of epithelial integrity

<table>
<tr><th></th><th>Day -2</th><th>Day -1</th><th>Day 0</th><th>Day 1</th></tr>
<tr><td>Rescue</td><td rowspan="2">mTeSR1
+ Ri</td><td rowspan="2">mTeSR1</td><td>mTeSR1
+ LPA (5 μM)</td><td>DE medium
+ ActivinA (100ng/mL)
+ LPA (5 μM)</td></tr>
<tr><td>Chemical induction</td><td>mTeSR1
+ Blebbistatin (10 μM)</td><td>DE medium
+ ActivinA (100ng/mL)
+ Blebbistatin (10 μM)</td></tr>
</table>

Supplementary Table 5. Medium replacement protocol for stimulation assays

	Day -2	Day -1	Day 0
**BMP**	mTeSR1 + Ri	mTeSR1	mTeSR1 + Bmp4 (50ng/mL) 1 hour
**ACTIVIN**			mTeSR1 + ActivinA (100ng/mL) 1 hour
**WNT**			mTeSR1 + Wnt3A (5, 10, 25 or 50ng/mL) 6 hours

Supplementary Table 6. List of human qPCR primers

Gene name	Gene full name	Primer sequence (fw/rv) 5'-3'
ACTR2B	activin A receptor type 2B	TGGGGTTTTGCTGCTTGAAC
		AACTGCTTCTTCACACAGGGG
BMP4	bone morphogenetic protein 4	GAAAGGGGCTTCCACCGTAT
		CGCAGGGCTCACATCAAAAG
BMPR1A	bone morphogenetic protein receptor type 1A	GCTCGTCTCTTGCCTTAGTGT
		TTAGGAACACACCTCCTCCCT
CER1	Cerberus 1	CCCGCATTTCCCAAAGCAAA
		CCCGCATTTCCCAAAGCAAA
CRIPTO	teratocarcinoma-derived growth factor 1	CGACCTTCTGGGGAAAACGA
		ATGCAGGTTCCCCCATTCAG
CXCR4	C-X-C Motif Chemokine Receptor 4	CACCGCATCTGGAGAACCA
		GCCCATTTCCTCGGTGTAGTT
EOMES	Eomesodermin	CACATTGTAGTGGGCAGTGG
		CGCCACCAAACTGAGATGAT
FOXA2	Forkhead box A2	GGAACACCACTACGCCTTCAAC
		AGTGCATCACCTGTTCGTAGGC
GAPDH	Glyceraldehyde 3-phosphate dehydrogenase	GTCTCCTCTGACTTCAACAGCG
		ACCACCCTGTTGCTGTAGCCAA
GPC4	Glypican4	GTCAGCGAACAGTGCAATCAT
		ACATTTCCCACCACGTAGTAAC
GSC	Gosecoid	GAGGAGAAAGTGGAGGTCTGGTT
		CTCTGATGAGGACCGCTTCTG
MESP1	Mesoderm Posterior BHLH Transcription Factor 1	TGAGGAGCCCAAGTGACAAG
		CCTGCTTGCCTCAAAGTGTC
MIXL1	Mix Paired-Like Homeobox 1	CCGAGTCCAGGATCCAGGTA
		CTCTGACGCCGAGACTTGG
NANOG	Nanog	CTCCAACATCCTGAACCTCAGC
		CGTCACACCATTGCTATTCTTCG
NODAL	Nodal Growth Differentiation Factor	CCGAGGGCAGACATCATCC
		CCATCCACTGCCACATCTTCT
OCT4	Octamer-binding transcription factor 4	CCTCACTTCACTGCACTGTA
		CAGGTTTTCTTTCCCTAGCT
OTX2	Orthodenticle homeobox 2	GGAAGCACTGTTTGCCAAGACC
		CTGTTGTTGGCGGCACTTAGCT
PDGFRa	Paleted derived growth factor receptor alpha	GTGATAATCCCCACAGGCACA
		ACATGAACAGGGGCATTCGT
SOX2	SRY (sex determining region Y)-box 2	GGCGAACCATCTCTGTGGTC
		TACCAACGGTGTCAACCTGC
SOX17	SRY-Box 17	CCGAGTTGAGCAAGATGCTG
		TGCATGTGCTGCACGCGCA
TBX6	T-Box Transcription Factor 6	AGCCTGTGTCTTTCCATC
		GCTGCCCGAACTAGGTGTAT
VEGFR2	Vascular endothelial growth factor receptor 2	CGGTCAACAAAGTCGGGAGA
		CAGTGCACCACAAAGACACG

Supplementary Table 7. Primaries antibodies used for Western-blot analyses

Antigen	Specie	Company	Reference	Dilution
ACTIN	Mouse	Sigma	A3853	1/10000
ACTR2B	Mouse	SantaCruz	sc-390977	1/100
AKT	Rabbit	Cell Signalling	9272	1/2000
AKT pS473	Rabbit	Cell Signalling	4060	1/2000
AKT pT308	Rabbit	Cell Signalling	13038	1/1000
BMPR1A	Mouse	SantaCruz	sc-518037	1/100
B-CATENIN	Rabbit	Cell Signalling	8480	1/2000
CRIPTO	Rabbit	Abcam	ab108391	1/2000
E-CADHERIN	Rabbit	Cell Signalling	3195	1/2000
ERK1,2,3	Mouse	Cell Signalling	9102	1/2000
ERK 1,2,3 pT202/Y204	Rabbit	Cell Signalling	9106	1/1000
GSK3 a/b	Rabbit	Cell Signalling	5676	1/1000
GSK3 a/b pS21/S9	Rabbit	Cell Signalling	9331	1/1000
NANOG	Rabbit	Cell Signalling	4903	1/4000
N-CADHERIN	Mouse	Thermofisher	333900	1/1000
OCCLUDIN	Mouse	Invitrogen	33-1500	1/500
OCT4	Rabbit	Cell Signalling	2840	1/4000
OTX2	Goat	R&D	967338	1/1000
SMAD2	Rabbit	Cell Signalling	5339	1/1000
SMAD2 pS465/S467	Rabbit	Cell Signalling	3108	1/1000
SOX2	Mouse	SantaCruz	sc365823	1/1000
ZO-1	Mouse	Thermofisher	339100	1/2000

Supplementary Table 8. List of Primaries antibodies used for immuno-cytochemical analyses

Antigen	Specie	Company	Reference	Dilution
ACTR2B	Mouse	SantaCruz	sc-390977	1/400
BMPR1A	Mouse	SantaCruz	sc-518037	1/50
BRACHYURY	Goat	R&D	#967332	1/80
BRACHYURY (Micropatterns)	Goat	R&D	AF2085	1/250
CLEAVED CASP3 (A175)	Rabbit	Cell Signalling	#9661	1/300
E-CADHERIN	Rabbit	Cell Signalling	#3195	1/200
EOMES	Rabbit	Abcam	ab23345	1/700
GPC4	Mouse	Genetex	GTX50007	1/500
HISTON H3 pS10	Rat	Millipore	06-570	1/250
LEF1	Rabbit	Cell Signalling	#2230	1/200
NA^{+} K^{+} ATPase A1	Rabbit	Cell Signalling	#23565	1/100
NANOG	Rabbit	Cell Signalling	#4903	1/200
N-CADHERIN	Mouse	Thermofisher	333900	1/200
OCCLUDIN	Mouse	Invitrogen	33-1500	1/500
OCT4	Rabbit	Cell Signalling	#2840	1/400
OTX2	Goat	R&D	#967338	1/80
PDGFRa	Rabbit	R&D	AF-307-NA	1/100
SMAD1,5 pS463/S465	Rabbit	Cell Signalling	#9516	1/300
SMAD2 pS465/S467	Rabbit	Thermofisher	44-244G	1/250
SOX2	Mouse	SantaCruz	sc365823	1/1000
SOX2 (Micropatterns)	Rat	Thermofisher	14-9811-82	1/500
SOX17	Goat	R&D	#967330	1/80
SOX17 (Micropatterns)	Goat	R&D Systems	AF1924	1:250
ZO-1	Mouse	Thermofisher	339100	1/500

Supplementary Table 9. List of Primaries antibodies used for FACS analyses

Antigen	Specie	Company	Reference	Dilution
EOMES APC-conjugated	Mouse	R&D	IC6166A	10 µL/10^6 cells
PDGFRA Monoclonal Antibody (16A1), APC	Mouse	Thermofisher	A15718	10 µL/10^6 cells
Mouse IgG2B APC-conjugated	Mouse	R&D	IC0041A	10 µL/10^6 cells
Fc-Receptors anti-CD32 (3D3)	Mouse	BD Bioscience	551900	0.5 mg/ml
