# Supplementary material for: Epithelial disruption drives mesendoderm differentiation in human pluripotent stem cells by enabling TGF-β protein sensing
Source: Nat Commun. 2023 Jan 21;14:349. doi: 10.1038/s41467-023-35965-8 (PMC9867713; doi:10.1038/s41467-023-35965-8)
Supplement: Supplementary file 3 — Reporting Summary [file 41467_2023_35965_MOESM3_ESM.pdf]

nature portfolio

Corresponding author(s): Rosanna Dono

Last updated by author(s): Jan 10, 2023

Reporting Summary

Nature Portfolio wishes to improve the reproducibility of the work that we publish. This form provides structure for consistency and transparency in reporting. For further information on Nature Portfolio policies, see our Editorial Policies and the Editorial Policy Checklist.

Statistics

For all statistical analyses, confirm that the following items are present in the figure legend, table legend, main text, or Methods section.

n/a	Confirmed	
☐	☒	The exact sample size (*n*) for each experimental group/condition, given as a discrete number and unit of measurement
☐	☒	A statement on whether measurements were taken from distinct samples or whether the same sample was measured repeatedly
☐	☒	The statistical test(s) used AND whether they are one- or two-sided<br>*Only common tests should be described solely by name; describe more complex techniques in the Methods section.*
☒	☐	A description of all covariates tested
☐	☒	A description of any assumptions or corrections, such as tests of normality and adjustment for multiple comparisons
☐	☒	A full description of the statistical parameters including central tendency (e.g. means) or other basic estimates (e.g. regression coefficient) AND variation (e.g. standard deviation) or associated estimates of uncertainty (e.g. confidence intervals)
☐	☒	For null hypothesis testing, the test statistic (e.g. *F*, *t*, *r*) with confidence intervals, effect sizes, degrees of freedom and *P* value noted<br>*Give P values as exact values whenever suitable.*
☒	☐	For Bayesian analysis, information on the choice of priors and Markov chain Monte Carlo settings
☒	☐	For hierarchical and complex designs, identification of the appropriate level for tests and full reporting of outcomes
☒	☐	Estimates of effect sizes (e.g. Cohen's *d*, Pearson's *r*), indicating how they were calculated

Our web collection on statistics for biologists contains articles on many of the points above.

Software and code

Policy information about availability of computer code

Data collection	Immunofluorescence: ZEISS AxioImager, LSM 780, ZEN lite blue edition, Olympus IX83 with ANDOR zyla camera and DSD2 spinning disk confocal system. QRT-PCR: StepOne (Applied Biosystems), CFX96 (Bio-Rad). FACS: BD LSRFortessa 5Laser SORP cytometer (BD Bioscience) .
Data analysis	RNA sequencing analyses: The quality of the raw reads was assessed using FastQC v0.11.9 toolkit (Andrews, 2010). Adapters and low-quality reads were trimmed using Trimmomatic v0.39 (Bolger et al., 2014). Paired-end reads were aligned with HiSat2 v2.2.1 (Kim et al., 2015) using default options. Gene counts were quantified using feature Counts v2.0.1 from the Subread v2.0.1 package (Liao et al., 2019). Gene counts were then normalized and used for differential expression testing using DESeq2 v1.28.038 (Supplementary Tables 1,2). The workflow allowing to achieve the differential expression analysis of RNA-seq data is available on Zenodo (https://zenodo.org/) with the DOI 10.5281/zenodo.5569383 and is under the BSD-3 license. For the differential RNA-seq analyses, the genes with a log2fc between -0.58 to 0.58 and an adjusted p-value (padj) > 0.01 were not considered as significantly differentially expressed.<br>Other programs: Prism (version 8.4.3), Zen Blue (version 3.4.9100000), Image J (version 2.3.051), matlab R21b, BD FACS DIVA 9.0.1 software, Cytobank 9.4 (Beckman Coulter).

For manuscripts utilizing custom algorithms or software that are central to the research but not yet described in published literature, software must be made available to editors and reviewers. We strongly encourage code deposition in a community repository (e.g. GitHub). See the Nature Portfolio guidelines for submitting code & software for further information.

Data

Policy information about availability of data

All manuscripts must include a data availability statement. This statement should provide the following information, where applicable:

- Accession codes, unique identifiers, or web links for publicly available datasets
- A description of any restrictions on data availability
- For clinical datasets or third party data, please ensure that the statement adheres to our policy

The data that support this study are available in the source data provided with this paper. For the RNA-seq alignment and gene counts were generated against the GRCh38.p13 (Ensembl release 101; IDs: 2334371 [UID] 8687898 [GenBank] 8765528 [RefSeq]). The RNA-seq data and the read count matrix are available on Zenodo (https://zenodo.org/) with the DOI 10.5281/zenodo.5569383. The RNA-seq data have also been deposited in NCBI's Gene Expression Omnibus and are accessible through GEO Series accession number GSE222186 (https://www.ncbi.nlm.nih.gov/geo/query/acc.cgi?acc=GSE222186).
Human induced pluripotent stem cell (hiPSC) lines used in this study are available at the WiCells (https://www.wicell.org/). HiPSCs with downregulated GPC4 levels are available from the authors.

Human research participants

Policy information about studies involving human research participants and Sex and Gender in Research.

Reporting on sex and gender	N/A
Population characteristics	N/A
Recruitment	N/A
Ethics oversight	N/A

Note that full information on the approval of the study protocol must also be provided in the manuscript.

Field-specific reporting

Please select the one below that is the best fit for your research. If you are not sure, read the appropriate sections before making your selection.

☒ Life sciences ☐ Behavioural & social sciences ☐ Ecological, evolutionary & environmental sciences

For a reference copy of the document with all sections, see nature.com/documents/nr-reporting-summary-flat.pdf

Life sciences study design

All studies must disclose on these points even when the disclosure is negative.

Sample size	Sample size calculations were not performed. Sample sizes were chosen as large as possible while taking into account the experimental effort required to generate the respective data and taking into account the most optimal numbers to generate statistically significant results. All experiments were repeated for at least three times (three biologically independent samples unless otherwise stated).
Data exclusions	No data were excluded
Replication	The number of biologically independent samples is reported in the figure legends. Experiments were successfully replicated in at least 2-3 independent studies.
Randomization	Cell linese were allocated according to: close passage numbers and GPC4 expression levels (RTqPCR and immunocytochemistry).
Blinding	Investigators were not blinded, but this was not necessary as data were collected and examined by different investigators.

Reporting for specific materials, systems and methods

We require information from authors about some types of materials, experimental systems and methods used in many studies. Here, indicate whether each material, system or method listed is relevant to your study. If you are not sure if a list item applies to your research, read the appropriate section before selecting a response.

Materials & experimental systems

n/a	Involved in the study
	☒ Antibodies
	☒ Eukaryotic cell lines
☒	Palaeontology and archaeology
☒	Animals and other organisms
☒	Clinical data
☒	Dual use research of concern

Methods

n/a	Involved in the study
☒	ChIP-seq
	☒ Flow cytometry
☒	MRI-based neuroimaging

Antibodies

Antibodies used

For Western-blot analyses: ACTIN; Mouse; Sigma; A3853; clone AC-40; 1/10000. ACTR2B; Mouse; SantaCruz ; sc-390977; clone F-12; 1/100. AKT; Rabbit; Cell Signalling; 9272; 1/2000. AKT pS473; Rabbit; Cell Signalling; 4060; clone D9E; 1/2000. AKT pT308; Rabbit; Cell Signalling; 13038; clone D25E6; 1/1000. BMPR1A; Mouse; SantaCruz ; sc-518037; clone C-7; 1/100. B-CATENIN; Rabbit; Cell Signalling; 8480; clone D10A8; 1/2000. CRIPTO; Rabbit; Abcam; ab108391; clone EPNCIR106A; 1/2000. E-CADHERIN; Rabbit; Cell Signalling; 3195; clone 24E10; 1/2000. ERK1,2; Mouse; Cell Signalling; 9102; 1/2000. ERK 1,2,3 pT202/Y204; Rabbit; Cell Signalling; 9106; clone E10; 1/1000. GSK3 a/b; Rabbit; Cell Signalling; 5676; clone D75D3; 1/1000. GSK3 a/b pS21/S9; Rabbit; Cell Signalling; 9331; 1/1000. NANOG; Rabbit; Cell Signalling; 4903; clone D73G4; 1/4000. N-CADHERIN; Mouse; Thermofisher; 333900; clone 3B9; 1/1000. OCCLUDIN; Mouse; Invitrogen; 33-1500; clone OC-3F10; 1/500. OCT4; Rabbit; Cell Signalling; 2840; clone C30A3; 1/4000. OTX2; Goat; R&D; 967338; discontinued; 1/1000. SMAD2; Rabbit; Cell Signalling; 5339; clone D43B4; 1/1000.SMAD2 pS465/S467; Rabbit; Cell Signalling; 3108; clone 138D4; 1/1000. SOX2; Mouse; SantaCruz; sc365823; clone E-4; 1/1000. ZO-1; Mouse; Thermofisher; 339100; clone ZO1-1A12; 1/2000.

For immuno-cytochemical analyses: ACTR2B; Mouse; SantaCruz ; sc-390977; clone F-12; 1/400. BMPR1A; Mouse; SantaCruz; sc-518037; clone C-7; 1/50. BRACHYURY; Goat; R&D; #967332; discontinued; 1/80. BRACHYURY (Micropatterns); Goat; R&D; AF2085; 1/250. CLEAVED CASP3 (A175); Rabbit; Cell Signalling. #9661; clone Asp175; 1/300. E-CADHERIN; Rabbit; Cell Signalling; #3195; clone 24E10; 1/200. EOMES; Rabbit; Abcam; ab23345; 1/700. GPC4; Mouse; Genetex; GTX50007; clone AT4A3; 1/500. HISTON H3 pS10; Rat; Millipore; 06-570; 1/250. LEF1; Rabbit; Cell Signalling; #2230; clone C12A5; 1/200. NA+ K+ ATPase A1; Rabbit; Cell Signalling; #23565; clone D4Y7E; 1/100. NANOG; Rabbit; Cell Signalling; #4903; clone D73G4; 1/200. N-CADHERIN; Mouse; Thermofisher; 333900; clone 3B9; 1/200. OCCLUDIN; Mouse; Invitrogen; 33-1500; clone OC-3F10; 1/500. OCT4; Rabbit; Cell Signalling; #2840; clone C30A3; 1/400. OTX2; Goat; R&D; #967338; discontinued; 1/80. PDGFRa; Rabbit; R&D; AF-307-NA; 1/100. SMAD1,5 pS463/S465; Rabbit; Cell Signalling; #9516; clone 41D10; 1/300. SMAD2 pS465/S467; Rabbit; Thermofisher; 44-244G; 1/250. SOX2; Mouse; SantaCruz; sc365823; clone E-4; 1/1000. SOX2 (Micropatterns); Rat; Thermofisher; 14-9811-82; clone Btjce; 1/500. SOX17; Goat; R&D; #967330; discontinued; 1/80.SOX17 (Micropatterns); Goat; R&D Systems; AF1924; 1:250.ZO-1; Mouse; Thermofisher; 339100; clone ZO1-1A12; 1/500.

For FACS analysesEOMES APC-conjugated; Mouse; R&D; IC6166A; 10 µL/106 cells. PDGFRA Monoclonal Antibody, APC; Mouse; Thermofisher; A15718; clone 16A1; 10 µL/10^6 cells. Mouse IgG2B APC-conjugated; Mouse; R&D; IC0041A; clone #133303; 10 µL/10^6 cells. Fc-Receptors anti-CD32; Mouse; BD Bioscience; 551900; clone 3D3; 0.5 mg/ml.

Validation

All antibodies were used according to the manufacturers' recommendations. Companies provide quality certificates and information about validation strategies. For this study, antibodies were first tested with different dilutions, and optimal dilution ratios used in the study are stated in supplementary tables 7-9. Different lots of antibodies were used. As lots can be variable each lot was verified in house to replicate the correct staining. Additionally, all primary and secondary antibodies have been tested to ensure there was no non-specific binding.

Companies quality certificates

For Western-blot analyses:
ACTIN; Mouse; Sigma; A3853; clone AC-40; https://www.sigmaaldrich.com/FR/fr/product/sigma/a3853
ACTR2B; Mouse; SantaCruz ; sc-390977; clone F-12; https://www.scbt.com/fr/p/actr-ii-antibody-f-12
AKT; Rabbit; Cell Signalling; 9272; https://www.cellsignal.com/products/primary-antibodies/akt-antibody/9272
AKT pS473; Rabbit; Cell Signalling; 4060; clone D9E; https://www.cellsignal.com/products/primary-antibodies/phospho-akt-ser473-d9e-xp-rabbit-mab/4060
AKT pT308; Rabbit; Cell Signalling; 13038; clone D25E6; https://www.cellsignal.com/products/primary-antibodies/phospho-akt-thr308-d25e6-xp-rabbit-mab/13038
BMPR1A; Mouse; SantaCruz ; sc-518037; clone C-7; https://www.scbt.com/fr/p/bmpr-ia-antibody-c-7
B-CATENIN; Rabbit; Cell Signalling; 8480; clone D10A8; https://www.cellsignal.com/products/primary-antibodies/b-catenin-d10a8-xp-rabbit-mab/8480
CRIPTO; Rabbit; Abcam; ab108391; clone EPNCIR106A; https://www.abcam.com/cripto1cripto-antibody-epncir106a-ab108391.html
E-CADHERIN; Rabbit; Cell Signalling; 3195; clone 24E10; https://www.cellsignal.com/products/primary-antibodies/e-cadherin-24e10-rabbit-mab/3195
ERK1,2; Mouse; Cell Signalling; 9102; 1/2000.
ERK 1,2,3 pT202/Y204; Rabbit; Cell Signalling; 9106; clone E10; https://www.cellsignal.com/products/primary-antibodies/p44-42-mapk-erk1-2-antibody/9102
GSK3 a/b; Rabbit; Cell Signalling; 5676; clone D75D3; https://www.cellsignal.com/products/primary-antibodies/gsk-3a-b-d75d3-rabbit-mab/5676
GSK3 a/b pS21/S9; Rabbit; Cell Signalling; 9331; https://www.cellsignal.com/products/primary-antibodies/gsk-3a-b-d75d3-rabbit-mab/5676

NANOG; Rabbit; Cell Signalling; 4903; clone D73G4; https://www.cellsignal.com/products/primary-antibodies/nanog-d73g4-xp-rabbit-mab/4903
N-CADHERIN; Mouse; Thermofisher; 333900; clone 3B9; https://www.thermofisher.com/antibody/product/N-cadherin-Antibody-clone-3B9-Monoclonal/33-3900
OCCLUDIN; Mouse; Invitrogen; 33-1500; clone OC-3F10; https://www.thermofisher.com/antibody/product/Occludin-Antibody-clone-OC-3F10-Monoclonal/33-1500OCT4; Rabbit; Cell Signalling; 2840; clone C30A3; 1/4000.
OTX2; Goat; R&D; 967338; https://www.rndsystems.com/products/human-otx2-antibody_af1979
SMAD2; Rabbit; Cell Signalling; 5339; clone D43B4; https://www.cellsignal.com/products/primary-antibodies/smad2-d43b4-xp-rabbit-mab/5339
SMAD2 pS465/S467; Rabbit; Cell Signalling; 3108; clone 138D4; https://www.cellsignal.com/products/primary-antibodies/phospho-smad2-ser465-467-138d4-rabbit-mab/3108
SOX2; Mouse; SantaCruz; sc365823; clone E-4; https://www.scbt.com/fr/p/sox-2-antibody-e-4
ZO-1; Mouse; Thermofisher; 339100; clone ZO1-1A12; https://www.thermofisher.com/antibody/product/ZO-1-Antibody-clone-ZO1-1A12-Monoclonal/33-9100
For immuno-cytochemical analyses:
ACTR2B; Mouse; SantaCruz ; sc-390977; clone F-12; https://www.scbt.com/fr/p/actr-ii-antibody-f-12
BMPR1A; Mouse; SantaCruz; sc-518037; clone C-7; https://www.scbt.com/fr/p/bmpr-ia-antibody-c-7
BRACHYURY; Goat; R&D; #967332; https://www.rndsystems.com/products/human-mouse-brachyury-antibody_af2085?gclid=EAIaIQobChMIrrn83Zz--wIV74xoCR2dkA80EAAYAiAAEgINXvD_BwE&gclsrc=aw.ds
BRACHYURY (Micropatterns); Goat; R&D; AF2085; clone # O15178; https://www.rndsystems.com/products/human-mouse-brachyury-antibody_af2085?gclid=EAIaIQobChMIm8yS75z--wIVBp_VCh3r8AQCEAAYASAAEgIsC_D_BwE&gclsrc=aw.ds
CLEAVED CASP3 (A175); Rabbit; Cell Signalling. #9661; clone Asp175; https://www.cellsignal.com/products/primary-antibodies/cleaved-caspase-3-asp175-antibody/9661E-CADHERIN; Rabbit; Cell Signalling; #3195; clone 24E10; 1/200.
EOMES; Rabbit; Abcam; ab23345; https://www.abcam.com/tbr2--eomes-antibody-ab23345.html
GPC4; Mouse; Genetex ; GTX50007; clone AT4A3; https://pr.vwr.com/store/product/16670996/anti-gpc4-mouse-monoclonal-antibody-clone-at4a3
HISTON H3 pS10; Rat; Millipore; 06-570; https://www.merckmillipore.com/FR/fr/product/Anti-phospho-Histone-H3-Ser10-Antibody-Mitosis-Marker,MM_NF-06-570
LEF1; Rabbit; Cell Signalling; #2230; clone C12A5; https://www.cellsignal.com/products/primary-antibodies/lef1-c12a5-rabbit-mab/2230
NA+ K+ ATPase A1; Rabbit; Cell Signalling; #23565; clone D4Y7E; https://www.cellsignal.com/products/primary-antibodies/na-k-atpase-a1-d4y7e-rabbit-mab/23565NANOG; Rabbit; Cell Signalling; #4903; clone D73G4; 1/200.
N-CADHERIN; Mouse; Thermofisher; 333900; clone 3B9; https://www.thermofisher.com/antibody/product/N-cadherin-Antibody-clone-3B9-Monoclonal/33-3900
OCCLUDIN; Mouse; Invitrogen; 33-1500; clone OC-3F10; https://www.thermofisher.com/antibody/product/Occludin-Antibody-clone-OC-3F10-Monoclonal/33-1500
OCT4; Rabbit; Cell Signalling; #2840; clone C30A3; https://www.cellsignal.com/products/primary-antibodies/oct-4a-c30a3-rabbit-mab/2840
OTX2; Goat; R&D; #967338; https://www.rndsystems.com/products/human-otx2-antibody_af1979PDGFRa; Rabbit; R&D; AF-307-NA; clone # P16234; 1/100.
SMAD1,5 pS463/S465; Rabbit; Cell Signalling; #9516; clone 41D10; https://www.cellsignal.com/products/primary-antibodies/phospho-smad1-5-ser463-465-41d10-rabbit-mab/9516
SMAD2 pS465/S467; Rabbit; Thermofisher; 44-244G; https://www.thermofisher.com/antibody/product/Phospho-SMAD2-Ser465-Ser467-Antibody-Polyclonal/44-244G
SOX2; Mouse; SantaCruz; sc365823; clone E-4; https://www.scbt.com/fr/p/sox-2-antibody-e-4SOX2 (Micropatterns); Rat; Thermofisher; 14-9811-82; clone Btjce; 1/500.
SOX17; Goat; R&D; #967330; https://www.rndsystems.com/products/human-sox17-antibody-614013_mab19241?gclid=EAIaIQobChMIjM_yrp7--wIVWojVCh2nRgQdEAAYASAAEgL4tfD_BwE&gclsrc=aw.ds
SOX17 (Micropatterns); Goat; R&D Systems; AF1924; clone # Q9H6I2; https://www.rndsystems.com/products/human-sox17-antibody_af1924
ZO-1; Mouse; Thermofisher; 339100; clone ZO1-1A12; https://www.thermofisher.com/antibody/product/ZO-1-Antibody-clone-ZO1-1A12-Monoclonal/33-9100
For FACS analyses
EOMES APC-conjugated; Mouse; R&D; IC6166A; clone # O95936; 10 µL/106 cells. https://www.rndsystems.com/products/human-eomes-apc-conjugated-antibody-644730_ic6166a
PDGFRA Monoclonal Antibody, APC; Mouse; Thermofisher; A15718; clone 16A1; 10 µL/10^6 cells. https://www.thermofisher.com/antibody/product/PDGFRA-Antibody-clone-16A1-Monoclonal/A15718
Mouse IgG2B APC-conjugated; Mouse; R&D; IC0041A; clone #133303; 10 µL/10^6 cells. https://www.rndsystems.com/products/mouse-igg2b-apc-conjugated-antibody_ic0041a
Fc-Receptors anti-CD32; Mouse; BD Bioscience; 551900; clone 3D3; 0.5 mg/ml. https://www.bdbiosciences.com/en-fr/products/reagents/flow-cytometry-reagents/research-reagents/single-color-antibodies-ruo/purified-mouse-anti-human-cd32.551900

Eukaryotic cell lines

Policy information about cell lines and Sex and Gender in Research

Cell line source(s)

AICS-0023 hiPSCs and WT 029 hiPSCs are available at WiCells (https://www.wicell.org/). Available from the authors the hiPSCs with downregulated GPC4 levels: AICS-0023 GPC4sh, GPC4sh5-c10, GPC4sh2-c3.

Authentication

All hiPSC lines used in this study were published in Corti S et al. Stem Cells Transl Med. 10(5):725-742 (2021) doi: 10.1002/sctm.20-0177, together with their full characterization. Moreover, we checked routinely for the expression of pluripotency markers such as OTX2, SOX2 and NANOG as well as for GPC4 downregulation levels by RTqPCR and immunocytochemistry.

Mycoplasma contamination	Mycoplasma contamination of all hiPSC lines was routinely tested using a PCR-based assay. The assay contained a PCR-negative control, a positive control and an internal control. All lines were mycoplasma negative.
Commonly misidentified lines (See ICLAC register)	None

Flow Cytometry

Plots

Confirm that:

☒ The axis labels state the marker and fluorochrome used (e.g. CD4-FITC).

☒ The axis scales are clearly visible. Include numbers along axes only for bottom left plot of group (a 'group' is an analysis of identical markers).

☒ All plots are contour plots with outliers or pseudocolor plots.

☒ A numerical value for number of cells or percentage (with statistics) is provided.

Methodology

Sample preparation	Undifferentiated or differentiated hiPSCs were isolated from monolayer cultures by enzymatic digestion using Accutase.
Instrument	BD LSR Fortessa Cell Analyzer.
Software	Acquisition Software: BD FACS DIVA 9.0.1.
Cell population abundance	All relevant cell populations illustrated on histograms represented above 93% of total cell events in each experimental conditions.
Gating strategy	All relevant cell events drawn were gated on Live Cells (FSC-A vs L/D), Cell Morphology (FSC-A vs SSC-A), Singlets FSC (FSC-A/FSC-H), Singlets SSC (SSC-A/SSC-H). A figure exemplifying the gating strategy is provided in the supplementary information.

☒ Tick this box to confirm that a figure exemplifying the gating strategy is provided in the Supplementary Information.
